# Identification and Validation of Iron Metabolism-Related Biomarkers in Endometriosis: A Mendelian Randomization and Single-Cell Transcriptomics Study

**DOI:** 10.3390/cimb47100831

**Published:** 2025-10-09

**Authors:** Juan Du, Zili Lv, Xiaohong Luo

**Affiliations:** School of Medicine and Life Sciences, Chengdu University of Traditional Chinese Medicine, Chengdu 611137, China; dujuan@cdutcm.edu.cn (J.D.);

**Keywords:** endometriosis, iron metabolism, biomarkers, mendelian randomization analysis, single-cell RNA sequencing

## Abstract

Studies have shown that the iron concentration in the peritoneal fluid of women is associated with the severity of endometriosis. Therefore, investigation of iron metabolism-related genes (IM-RGs) in endometriosis holds significant implications for both prevention and therapeutic strategies in affected patients. Differentially expressed IM-RGs (DEIM-RGs) were identified by intersecting IM-RGs with differentially expressed genes derived from GSE86534. Mendelian randomization analysis was employed to determine DEIM-RGs causally associated with endometriosis, with subsequent verification through sensitivity analyses and the Steiger test. Biomarkers associated with IM-RGs in endometriosis were validated using expression data from GSE86534 and GSE105764. Functional annotation, regulatory network construction, and immunological profiling were conducted for these biomarkers. Single-cell RNA sequencing (scRNA-seq) (GSE213216) was utilized to identify distinctively expressed cellular subsets between endometriosis and controls. Experimental validation of biomarker expression was performed via reverse transcription–quantitative polymerase chain reaction (RT-qPCR). BMP6 and SLC48A1, biomarkers indicative of cellular BMP response, were influenced by a medicus variant mutation that inactivated PINK1 in complex I, concurrently enriched by both biomarkers. The lncRNA NEAT1 regulated BMP6 through hsa-mir-22-3p and hsa-mir-124-3p, while SLC48A1 was modulated by hsa-mir-423-5p, hsa-mir-19a-3p, and hsa-mir-19b-3p. Immune profiling revealed a negative correlation between BMP6 and monocytes, whereas SLC48A1 displayed a positive correlation with activated natural killer cells. scRNA-seq analysis identified macrophages and stromal stem cells as pivotal cellular components in endometriosis, exhibiting altered self-communication networks. RT-qPCR confirmed elevated expression of BMP6 and SLC48A1 in endometriosis samples relative to controls. Both BMP6 and SLC48A1 were consistently overexpressed in endometriosis, reinforcing their potential as biomarkers. Moreover, macrophages and stromal stem cells were delineated as key contributors. These findings provide novel insights into therapeutic and preventive approaches for patients with endometriosis.

## 1. Introduction

Endometriosis is defined by the migration and implantation of viable endometrial tissue, including stroma and glands, at ectopic locations outside the uterine cavity, resulting in dysmenorrhea, severe pelvic pain, infertility, and psychological disorders such as anxiety, depression, and sleep disturbances, which collectively impair the physical and mental health of women of reproductive age [1,2]. The precise prevalence of endometriosis remains unclear, with estimates ranging from 2% to 10% in the general female population and reaching nearly 50% among women affected by infertility [3]. Despite its high burden, this disorder is frequently overlooked or underestimated [4]. The latency between the onset of symptoms and a definitive diagnosis may extend from 4 to 10 years, during which delayed recognition exacerbates individual suffering, perpetuates poor health, and results in a disease state that is more challenging to treat effectively [5].

Biomarkers are biological or physiological indicators reflecting normal or pathological processes or therapeutic responses, functioning as essential tools for diagnosis, prognosis, prediction, and drug evaluation [6]. For diagnostic purposes, disease biomarkers are principally categorized into nucleic acids and proteins [7]. The trajectory of biomarker discovery has shifted from morphological and cytogenetic markers to sophisticated molecular techniques such as polymerase chain reaction and next-generation sequencing, markedly enhancing diagnostic accuracy and facilitating targeted therapeutic strategies [8]. Currently, no single biomarker, nor any biomarker panel, has demonstrated sufficient accuracy and reliability for the definitive diagnosis of endometriosis [9]. Therefore, investigation into biomarkers for endometriosis holds substantial importance for promoting early diagnosis and timely intervention, thereby offering a pivotal pathway toward improved patient outcomes.

Ferroptosis, a regulated form of cell death closely linked to iron overload, is induced by hemosiderin deposition resulting from hemorrhage at ectopic endometriosis lesions, thereby initiating aberrant ferroptosis that subsequently influences cellular clearance [10]. Iron metabolism represents a central molecular mechanism governing ferroptosis [11]. In recent years, dysregulated iron metabolism has emerged as a defining feature of endometriosis, distinguishing it from other pathological conditions [12]. These findings highlight the importance of evaluating iron metabolism in women with endometriosis, indicating its potential as a valuable marker for both disease presence and progression. This perspective offers novel implications for endometriosis diagnosis and monitoring [13].

Mendelian randomization (MR) employs genetic variation to strengthen causal inference regarding modifiable risk factors for disease, relying on germline genetic variants to elucidate the impact of modifiable factors on outcomes [14]. To establish causal relationships between exposures and outcomes, MR typically applies genetic variants that satisfy the assumptions of an instrumental variable (IV), with single-nucleotide polymorphisms (SNPs) serving as the preferred instruments due to their abundance [15]. The core assumptions include a strong association between SNPs and relevant exposures, independence of SNPs from potential confounders, and the requirement that SNPs influence outcomes exclusively through the exposure of interest, thereby excluding alternative pathways. These principles ensure both methodological rigor and interpretability of MR analyses [16]. This approach has been utilized to investigate associations between endometriosis and epithelial abnormalities, ovarian cancer, and related conditions [17,18]. Moreover, Yan et al. reported that plasma ADAMTS13 expression was significantly correlated with endometriosis through two-sample MR analysis, suggesting its potential as a biomarker for endometriosis [19]. Collectively, these findings indicate that MR represents an important strategy for the identification of endometriosis-related biomarkers.

By integrating the robust causal inference afforded by MR with the unique resolution of single-cell analysis, in contrast to conventional transcriptomic methods, this study was designed to advance the identification and validation of iron metabolism-related biomarkers in endometriosis.

Given the emerging evidence that iron metabolism influences the pathophysiology of endometriosis, we propose that iron metabolism–related genes (IM-RGs) may be implicated in disease mechanisms and could represent potential diagnostic markers. To test this hypothesis, the present study integrates Mendelian randomization with single-cell transcriptomic analysis to identify and validate IM-RGs with a causal relationship to endometriosis.

## 2. Materials and Methods

### 2.1. Data Collection

The datasets GSE86534, GSE105764, and GSE213216 were obtained from the Gene Expression Omnibus database (https://www.ncbi.nlm.nih.gov/geo/ accessed on 19 January 2024). GSE86534 comprised microarray data from the GPL20115 platform, including tissue samples of 4 ectopic endometrium (EC) from endometriosis and 4 eutopic endometrium (EU) from controls [20]. GSE105764, derived from the GPL20301 platform for high-throughput analysis, contained tissue samples of 8 EC from endometriosis and 8 EU from controls, with only mRNA data selected [21]. GSE213216 was a single-cell RNA sequencing (scRNA-seq) dataset from the GPL24676 platform, consisting of tissue samples from 22 EC of endometriosis and 9 EU of controls [22]. GSE86534 and GSE105764 were used as the training and validation sets, respectively. Additionally, 505 iron metabolism-related genes (IM-RGs) were retrieved from the Molecular Signatures Database (MSigDB) (https://www.gsea-msigdb.org/gsea/index.jsp accessed on 19 January 2024) (Table S1). The dataset ukb-b-10903 for endometriosis (outcomes) was obtained from the Integrative Epidemiology Unit (IEU) Open Genome-wide Association Study (GWAS) database (https://gwas.mrcieu.ac.uk/ accessed on 28 January 2024). The GWAS dataset for endometriosis included 462,933 samples (3809 endometriosis cases and 459,124 controls) and 9,851,867 SNPs. All samples in this dataset were of European descent.

### 2.2. Identification and Enrichment Analyses of Differentially Expressed IM-RGs (DEIM-RGs)

Differential expression analysis was performed on endometriosis and control samples from the training set using “limma” (version 3.56.2), applying the thresholds |log2FoldChange (FC)| > 0.5 and *p* < 0.05 [23]. The results were visualized with “ggplot2” (version 3.4.2) and “pheatmap” (version 1.0.12) (https://CRAN.R-project.org/package=pheatmap accessed on 28 January 2024) to generate a volcano plot and heatmap, respectively [24]. DEIM-RGs were subsequently identified through the intersection of differentially expressed genes (DEGs) and the 505 IM-RGs by employing “ggVennDiagram” (version 1.2.3) [25]. To further investigate the biological processes associated with DEIM-RGs, Gene Ontology (GO) and Kyoto Encyclopedia of Genes and Genomes (KEGG) enrichment analyses were carried out (*p* < 0.05) using “clusterProfile” (version 4.8.2) [26].

### 2.3. Screening of Instrumental Variables (IVs) in MR Analysis

Expression quantitative trait loci data for DEIM-RGs (exposure factors) were obtained from the IEU OpenGWAS database. The extract instrument function of “TwoSampleMR” (version 0.5.8) was employed to read the exposure factors and filter SNPs, applying a threshold of *p* < 5 × 10^−8^ [27]. To ensure SNP independence, those in linkage disequilibrium (LD) were excluded using clump = TRUE, r^2^ = 0.001, and kb = 10,000. The outcome data were retrieved with the extract outcome data function of “TwoSampleMR” (version 0.5.8). Among the SNPs associated with exposure factors, variants unrelated to the outcome were removed. Furthermore, SNPs with F-statistic < 10 or with fewer than three instruments were excluded. The formula for calculating the F-statistic is $F=\frac{R^{2}}{1-R^{2}}\times \frac{N-K-1}{K}$, R^2^ represents the coefficient of determination, N denotes the sample size, and k indicates the number of included SNPs [28].

### 2.4. Mendelian Randomization (MR) Analysis and Expression Verification

To identify DEIM-RGs causally associated with endometriosis, uniform effect alleles and effect sizes were harmonized using the harmonize data function of “TwoSampleMR” (version 0.5.8). MR analysis was subsequently conducted with the mr function, integrating five algorithms [MR Egger, weighted median, inverse variance weighted (IVW), simple mode, weighted mode] [29,30,31,32,33]. Particular emphasis was placed on the IVW algorithm. The *p*-value, odds ratio (OR), and 95% confidence interval (95% CI) derived from the IVW method were evaluated, with *p* < 0.05 indicating a significant causal relationship between DEIM-RGs and endometriosis. Moreover, OR > 1 suggested that DEIM-RGs acted as risk factors for endometriosis, whereas OR < 1 indicated protective effects. DEIM-RGs identified through IVW and exhibiting consistent expression trends in the training set were defined as candidate genes. Scatter plots were applied to depict correlations between candidate genes and endometriosis, forest plots to illustrate SNP effect sizes on endometriosis for each candidate gene (evaluated by IVW), and funnel plots to assess the randomization of MR analysis. To evaluate the robustness of MR results, sensitivity analyses were conducted, including heterogeneity testing, horizontal pleiotropy testing, and leave-one-out (LOO) analysis. A *p*-value of Cochran’s Q test > 0.05 for IVW indicated no heterogeneity between candidate genes and endometriosis samples, whereas *p* > 0.05 in MR-Egger regression suggested no horizontal pleiotropy. LOO analysis confirmed the reliability of the overall effect in the absence of serious bias distortion. To exclude confounding by reverse causality, the Steiger test was performed, with TRUE and *p* < 0.05 regarded as evidence of causal direction. Finally, expression levels of candidate genes were analyzed in endometriosis and control samples from the training and validation sets using the Wilcoxon test (*p* < 0.05). Candidate genes with consistent expression trends and significant differences were designated as biomarkers for subsequent analyses.

### 2.5. Functional Annotation of Biomarkers

To identify additional genes functionally related to the biomarkers and their associated roles, the biomarkers were uploaded to the Gene Multiple Association Network Integration Algorithm (GeneMANIA) (https://genemania.org/ accessed on 21 February 2024) to construct the gene-gene interaction network (false discovery rate < 0.05). The reference gene set c2.cp.kegg.v2023.1.Hs.symbols.gmt was obtained from MSigDB (https://www.gsea-msigdb.org/gsea/msigdb/ accessed on 21 February 2024). With the biomarkers as the target genes, the correlation coefficients between the expression of all genes and the target genes were calculated in the training set, and the genes were ranked accordingly (descending order). Based on these rankings, gene set enrichment analysis (GSEA) was performed (*p* < 0.05) using “clusterProfiler” (version 4.8.2).

### 2.6. Construction of Regulatory Network

To investigate the molecular regulatory mechanisms of biomarkers, their miRNAs were predicted using miRTarBase (https://mirtarbase.cuhk.edu.cn/~miRTarBase/miRTarBase_2025/php/index.php accessed on 28 February 2024) and TarBase (https://dianalab.e-ce.uth.gr/tarbasev9 accessed on 28 February 2024) through the NetworkAnalyst platform (https://www.networkanalyst.ca/NetworkAnalyst/home.xhtml accessed on 28 February 2024). The intersection of the miRNAs predicted from both databases was defined as the targeted miRNAs of the biomarkers. Subsequently, starBase (https://rnasysu.com/encori/ accessed on 6 March 2024) was employed to predict the lncRNAs of the targeted miRNAs, and the lncRNA-miRNA-mRNA regulatory network was constructed using “Cytoscape” (version 3.10.0) [34].

### 2.7. Immune Infiltration Analysis

In the training set, to assess differences in the immune microenvironment between endometriosis and control samples, CIBERSORT was applied to estimate the composition and relative abundance of 22 immune cell types in the training set [35,36]. CIBERSORT is a computational method based on gene expression deconvolution. Its core principle involves utilizing the Support Vector Regression algorithm to fit the global gene expression profile of mixed tissues with a pre-defined signature gene set (Signature Matrix), thereby estimating the relative proportions of specific immune cell types within the mixed tissues. CIBERSORT commonly employs the LM22 signature gene set, which is designed to accurately distinguish 22 subtypes of human immune cells. The classification of these cell subtypes is based on their functions and lineages, specifically including:

B cells: naive B cells, memory B cells, plasma cells;

T cells: CD8^+^ T cells, naive CD4^+^ T cells, resting memory CD4^+^ T cells, activated memory CD4^+^ T cells, follicular helper T cells, regulatory T cells (Tregs), gamma delta T cells (γδ T cells);

Natural killer (NK) cells: resting NK cells, activated NK cells;

Myeloid cells: monocytes, M0 macrophages, M1 macrophages, M2 macrophages, resting dendritic cells, activated dendritic cells, resting mast cells, activated mast cells;

Granulocytes: eosinophils, neutrophils.

Infiltration levels of immune cells between endometriosis and controls were then compared using the Wilcoxon test (*p* < 0.05). Furthermore, correlations between biomarkers and differential immune cells were evaluated by Spearman analysis (|cor| > 0.5, *p* < 0.05).

### 2.8. ScRNA-Seq Data Analysis

In the GSE213216 dataset, “Seurat” (version 4.4.0) was employed for scRNA-seq data analysis [37]. Parameters were set as min.cells = 3 and min.features = 500 to generate the “Seurat” library. Double-cell detection was performed using “scDblFinder” (version 1.16.0), with the double-cell rate set to 8% for samples containing more than 10,000 cells and to 5% for those with fewer than 10,000 cells [38]. The screening criteria included library size > 200 and <95% of the second quartile, gene counts < 95% of the second quartile, mitochondrial content < 10%, and exclusion of bicellular cells. Following quality control, the FindVariableFeatures function was applied to select the top 2000 highly variable genes. All samples were integrated using IntegrateData, and principal component analysis (PCA) was conducted on cell distributions based on the 2000 highly variable genes. The percentage of variance explained by each principal component (PC) was ranked, and PCs preceding the elbow point in the PCA elbow plot were selected for downstream analysis. Subsequently, the FindNeighbors and FindClusters functions of “Seurat” (version 4.4.0) were used for unsupervised clustering of cells via UMAP (resolution = 1). Cell clusters were annotated according to marker genes obtained from the literature (Table S2) to identify distinct subpopulations [39]. Differences in cell subpopulations between endometriosis and control samples were evaluated using the Wilcoxon test (*p* < 0.05), and significantly altered subpopulations were designated as key cells. Visualization was performed with “ggplot2” (version 3.4.2). Cell–cell communication among subpopulations was analyzed with “CellChat” (version 1.6.1) to examine intercellular interactions [40]. Pseudo-time analysis of key cells was carried out using the Monocle2 algorithm implemented in “monocle” (version 2.30.0) to investigate cellular differentiation trajectories [41]. Finally, key cells were further subdivided into distinct subtypes by UMAP, and gene set variation analysis (GSVA) of these subtypes was conducted using “GSVA” (version 1.50.0) [42].

### 2.9. Reverse Transcription-Quantitative Polymerase Chain Reaction (RT-qPCR)

There are ethical and practical difficulties in obtaining fresh human endometriosis lesion tissue and matching normal endometrial tissue, as well as difficulty in controlling confounding factors. Therefore, to preliminarily verify the expression trend of biomarkers in the in vivo environment, this study chose to perform RT-qPCR in a rat model. An endometriosis model was generated in five SPF-grade female Sprague-Dawley rats (190–210 g) via autologous endometrial implantation. Under aseptic conditions, endometrial tissue fragments of 5 mm × 5 mm size were grafted into subfascial tunnels with the mucosal surface of muscle. Diethylstilbestrol (0.02 mg/kg) was administered orally for three days postoperatively. Lesions were evaluated every three days by palpation. After four weeks, laparotomy confirmed successful modeling by lesion volume (≥8 mm^3^), fluid-filled cysts (≥2 mm), and fibrotic encapsulation with neovascularization. Autologous endometrial fragments were observed to successfully adhere and implant at the grafting sites, forming cystic and fibrotic nodules resembling human endometriotic lesions. Histological confirmation was performed using hematoxylin and eosin (H&E) staining, which demonstrated preserved endometrial glandular epithelium and surrounding stromal components, thereby verifying the presence of ectopic endometrial tissue (Figure S1). Ectopic lesions were then harvested for subsequent analyses. Five sham-operated rats, which did not receive endometrial transplantation, served as controls.

To minimize variability, rats were age- and weight-matched, housed under standardized environmental conditions, and randomly assigned to groups. All animals underwent standardized surgical procedures and postoperative care, and outcome assessments were performed in a blinded manner to ensure reliability. For RT-qPCR validation of biomarkers, tissue samples were obtained from five ectopic lesions (EC group) in endometriosis model rats and five eutopic endometrial tissues (EU group) from sham-operated controls. Each sample was subjected to three technical repetitions. All procedures involving animals were conducted in accordance with institutional guidelines.

Total RNA was extracted from endometriosis and control tissues using TRIzol reagent. The RNA concentration and purity were measured with the NanoPhotometer N50. The RNA was reverse-transcribed into complementary DNA (cDNA) using the SweScript First Strand cDNA synthesis kit (Servicebio, Wuhan, China). qPCR was subsequently performed according to the manufacturer’s instructions. The amplification protocol was set as follows: 95 °C for 1 min, 95 °C for 20 s, 55 °C for 20 s, and 72 °C for 30 s. Relative gene expression levels were calculated using the 2^−△△CT^ method, with *GAPDH* serving as the internal reference gene. Primers were synthesized by Tsingke Biotech (Beijing, China) (Table S3).

### 2.10. Statistical Analysis

R software (version 4.2.2) was used to conduct all analyses. The linear model framework within the R package “limma”(version 3.56.2) was utilized to identify DEGs. The Wilcoxon test was used for intergroup comparisons in the analysis of gene expression and immune cell infiltration based on GEO datasets. For the Mendelian randomization analysis, the inverse variance weighting (IVW) method was adopted as the primary analytical approach, supplemented by a variety of methods for sensitivity analysis. A *p*-value less than 0.05 was regarded as statistically significant. In the GSE86534 dataset, 4 samples of EC tissue and 4 samples of EU tissue were derived from the same 4 patients. Similarly, in the GSE105764 dataset, 8 EC tissue samples and 8 EU tissue samples were obtained from the same 8 patients. This is used to control for inter-individual variability. For the RT-qPCR experiments, a two-tailed *t*-test was used for intergroup comparisons, and a *p*-value < 0.05 was considered statistically significant.

## 3. Results

### 3.1. Screening and GO/KEGG Enrichment Analysis of DEIM-RGs

**Figure 1:**
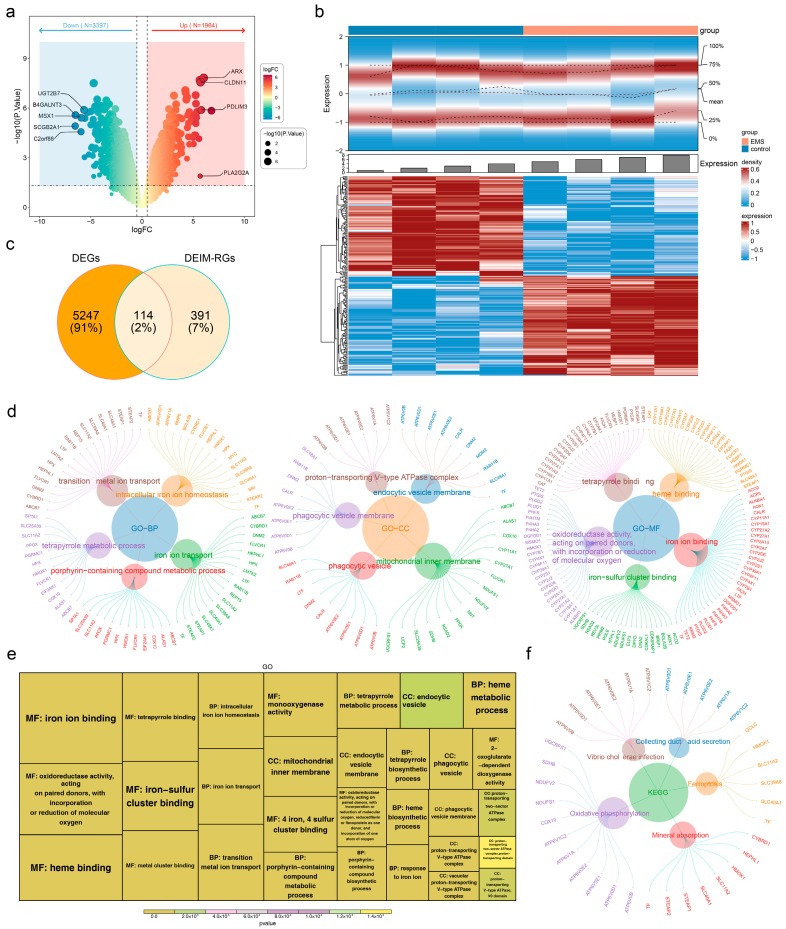
Screening of DEIM-RGs and GO/KEGG enrichment analysis. (**a**,**b**) Volcano and heat maps of DEGs in treatment and control samples from the GSE86534 dataset. (**c**) Venn diagram showing the overlap between DEGs and IM-RGs. (**d**,**e**) GO and KEGG enrichment analyses of DEIM-RGs. (**f**) Top five KEGG pathways enriched by DEIM-RGs.

In the endometriosis and control samples of the training set, 5361 DEGs were identified, including 1964 upregulated and 3397 downregulated genes (Figure 1a,b). From the intersection of these 5361 DEGs with 505 IM-RGs, 114 DEIM-RGs were obtained (Figure 1c). These 114 DEIM-RGs were significantly enriched in 527 GO terms, comprising 448 biological processes (BPs), 39 cellular components (CCs), and 40 molecular functions (MFs), along with 13 KEGG pathways. The top five and top ten BPs, CCs, and MFs were presented separately (Figure 1d,e), and the top five KEGG pathways were also displayed (Figure 1f). Notably, the 114 DEIM-RGs were predominantly enriched in iron ion transport (BP), phagocytic vesicle (CC), iron ion binding (MF), and oxidative phosphorylation (KEGG). Several enriched pathways, including phagocytic vesicle, oxidative phosphorylation, and heme binding, were closely related to endometriosis [43,44,45,46].

### 3.2. BMP6 and SLC48A1 Were Identified as Biomarkers

To identify genes causally associated with endometriosis, MR analysis was conducted. The results demonstrated that *BMP6* [OR = 1.001002221, 95% confidence interval (CI) = 1.00001099–1.001994429, *p* < 0.05] and *SLC48A1* (OR = 1.000723176, 95%CI = 1.000012434–1.00143442, *p* < 0.05) were significantly associated with the risk of endometriosis, suggesting that increased levels of these two genes were associated with an elevated risk of endometriosis and that they acted as risk factors for endometriosis according to the IVW algorithm, consistent with their expression trends in the training set (Table 1 and Table S1).

**Figure 2:**
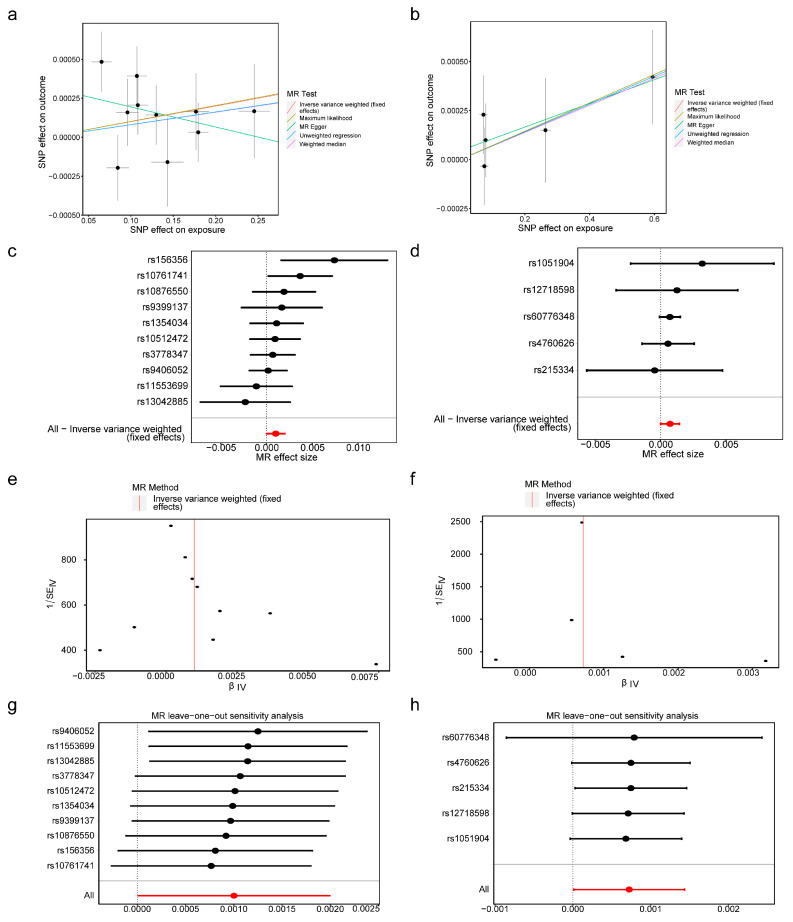
The biomarkers associated with endometriosis. (**a**,**b**) The scatter plots of *BMP6* and *SLC48A1* with endometriosis. (**c**,**d**) Forest plots of *BMP6* and *SLC48A1*. The label at the bottom of the figure, “All—Inverse variance weighted (fixed effects)”, represents the overall Mendelian randomization (MR) analysis result obtained by combining the effects of all single nucleotide polymorphisms (SNPs) using the fixed - effect inverse variance - weighted (IVW) method. The red dot indicates the total IVW result, and the line segment represents the 95% confidence interval. (**e**,**f**) The relationship between SNPs and IVW. The red line represents the effect-size regression line, which visually illustrates the overall trend of how effect sizes vary with their standard errors. (**g**,**h**) LOO analysis of *BMP6* and *SLC48A1*. 95% Confidence Interval for the total effect.

In scatter plots, the positive slopes of the IVW algorithm indicated that *BMP6* and *SLC48A1* increased endometriosis risk (Figure 2a,b). In forest plots, the overall effect sizes of SNPs for *BMP6* and *SLC48A1* were positioned to the right of zero, further suggesting their contribution to endometriosis susceptibility (Figure 2c,d). SNPs were approximately symmetrically distributed on both sides of IVW (Figure 2e,f), consistent with random allocation as described by Mendel’s second law.

The reliability of the MR results was supported by sensitivity analyses, including heterogeneity testing, horizontal pleiotropy testing, and LOO analysis. Cochran’s Q test showed *p* > 0.05 for *BMP6* and *SLC48A1* in the IVW heterogeneity test (Table 2). Similarly, MR-Egger regression yielded *p* > 0.05 for both genes in the pleiotropy test (Table 3). LOO analysis revealed no significant bias for *BMP6* or *SLC48A1* (Figure 2g,h), indicating robust MR results. Furthermore, the Steiger test was TRUE for both genes, demonstrating the absence of reverse causality between *BMP6*, *SLC48A1*, and endometriosis (Table 4). The exposure levels of BMP6 and SLC48A1 were shown to affect endometriosis, while endometriosis did not exert a reverse effect on the expression of BMP6 and SLC48A1.

**Figure 3:**
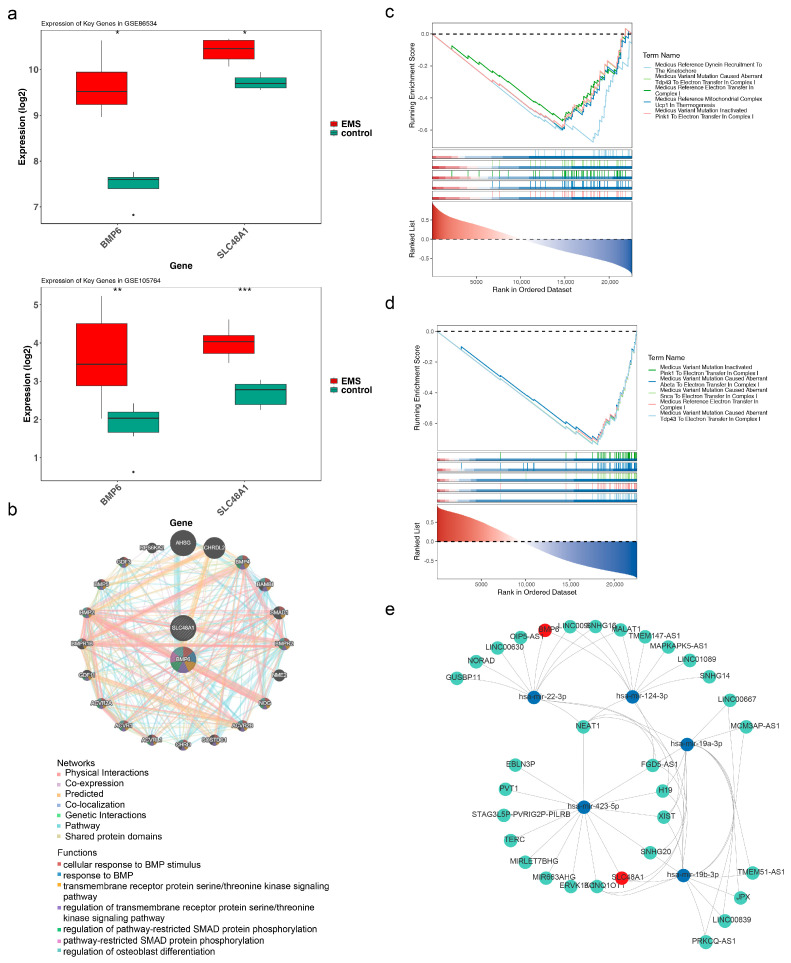
GeneMANIA and GSEA for *BMP6*, *SLC48A1,* and a network of lncRNA–miRNA–mRNA. (**a**) Expression of *BMP6* and *SLC48A1* in endometriosis and control samples from the training and validation sets. * *p* < 0.05. (**b**) Biological pathways associated with biomarkers. (**c**,**d**) KEGG pathways are significantly enriched for *BMP6* and *SLC48A1*. ** *p* < 0.01, *** *p* < 0.001. (**e**) Molecular regulatory network of *BMP6* and *SLC48A1*.

*BMP6* and *SLC48A1* were significantly upregulated in endometriosis samples compared with controls in both the training and validation sets (*p* < 0.05) (Figure 3a). Therefore, *BMP6* and *SLC48A1* were defined as biomarkers for subsequent analyses.

### 3.3. GeneMANIA and GSEA for BMP6 and SLC48A1

To explore biological pathways associated with the biomarkers, the top 20 genes (e.g., *AHSG*, *CHRDL2*, *BMP4*) functionally similar to *BMP6* and *SLC48A1* were identified. These genes were mainly involved in cellular response to BMP stimulus, response to BMP, and the transmembrane receptor protein serine/threonine kinase signaling pathway (Figure 3b). To further investigate pathways significantly related to the biomarkers, GSEA was performed. *BMP6* and *SLC48A1* were significantly enriched in 79 and 112 KEGG pathways, respectively, with the top five pathways displayed separately (Figure 3c,d). Among these, 65 KEGG pathways were concurrently enriched by both *BMP6* and *SLC48A1*, including medicus variant mutation–inactivated PINK1 to electron transfer in complex I and medicus variant mutation–induced aberrant abeta to electron transfer in complex I (Table S5). Enrichment results were primarily associated with complex I–related electron transfer pathways, which are frequently linked to oxidative processes [47,48,49,50]. These findings suggested that *BMP6* and *SLC48A1* may influence endometriosis through modulation of oxidation-related pathways.

### 3.4. Network of lncRNA-miRNA-mRNA

To further elucidate the association of biomarkers with endometriosis, their molecular regulatory mechanisms were investigated. Prediction analysis indicated that *BMP6* and *SLC48A1* were targeted by 2 and 3 miRNAs, respectively. In total, 57 lncRNAs were predicted for the five miRNAs, leading to the construction of an lncRNA–miRNA–mRNA regulatory network comprising 37 nodes and 62 edges (Figure 3e). Notably, NEAT1 regulated *BMP6* via hsa-mir-22-3p and hsa-mir-124-3p, while NEAT1 regulated *SLC48A1* through hsa-mir-423-5p, hsa-mir-19a-3p, and hsa-mir-19b-3p.

### 3.5. The Roles of BMP6 and SLC48A1 in the Immune Microenvironment Might Be Consistent

**Figure 4:**
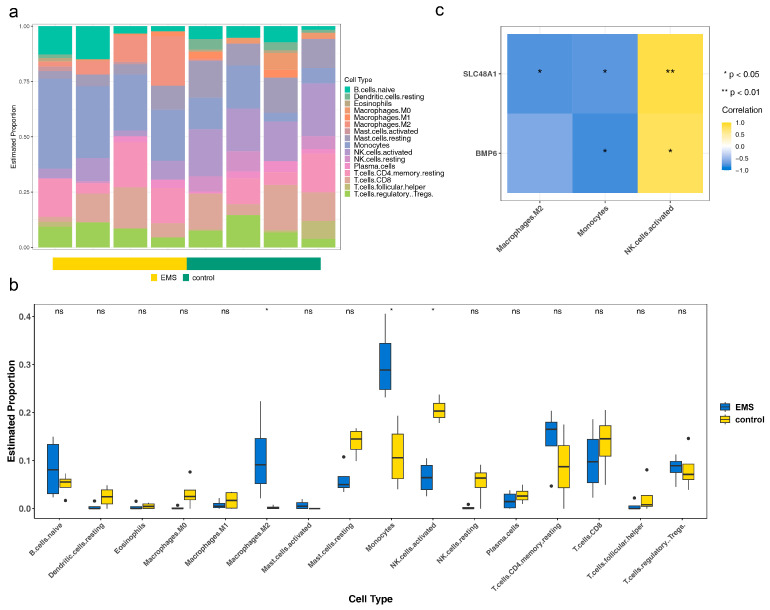
Association of *BMP6* and *SLC48A1* with immune microenvironment characteristics in endometriosis. (**a**) Immune infiltration analysis in endometriosis and control samples. (**b**) Relative abundance of immune cells in endometriosis and control samples. Ns not significant, * *p* > 0.05. (**c**) Correlations of *BMP6* and *SLC48A1* with the three differential immune cell types.

To investigate the immunological mechanisms of endometriosis, immune infiltration analysis was performed. The relative abundance of 19 immune cell types in endometriosis and control samples was assessed after excluding three cell types with zero abundance across all samples (Figure 4a). M2 macrophages and monocytes exhibited higher levels in endometriosis samples, while activated NK cells were more abundant in control samples, with all differences reaching statistical significance (*p* < 0.05) (Figure 4b). The correlations of *BMP6* and *SLC48A1* with these three differential immune cell types were consistent (Figure 4c). The strongest negative correlation was observed between *BMP6* and monocytes (cor = −0.81, *p* = 0.02), whereas the strongest positive correlation was found between *SLC48A1* and activated NK cells (cor = −0.86, *p* = 0.01) (Table S6). These findings suggested that monocytes and NK cells influence endometriosis progression, and *BMP6* and *SLC48A1* may contribute to disease development by modulating these immune cell populations [51,52,53,54].

### 3.6. Macrophages and Stromal Stem Cells Were Designated as Key Cells

**Figure 5:**
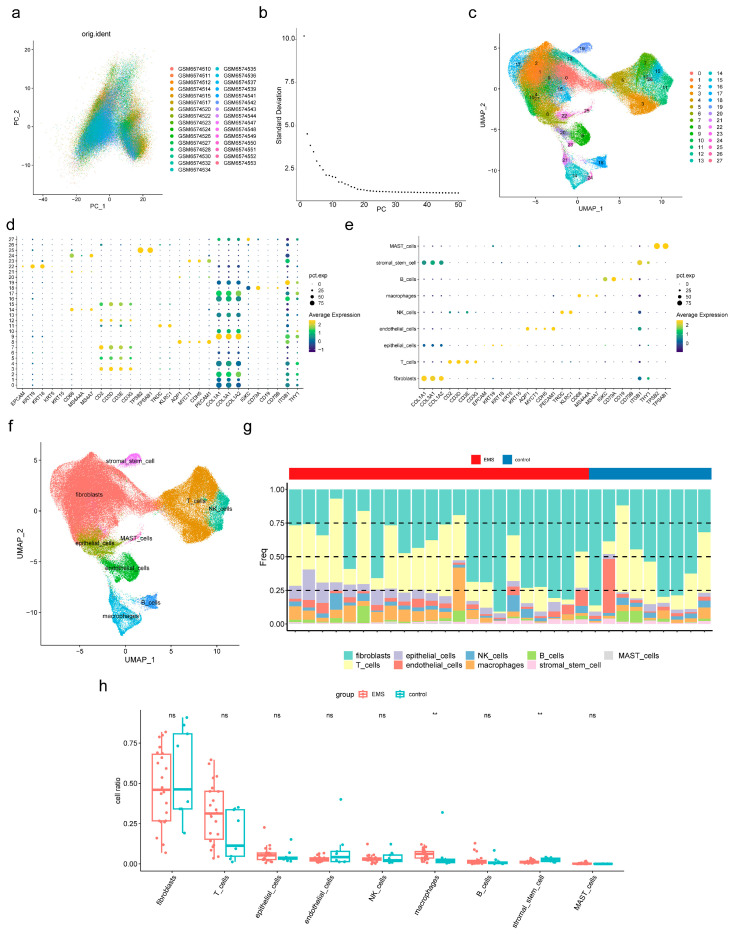
scRNA-seq analysis of key cells associated with endometriosis. (**a**) Scatter plot of principal component analysis (PCA) for single-cell transcriptome data, showing the distribution of cells after dimensionality reduction and the heterogeneity among samples. (**b**) This is a PCA scree plot, which assists in determining the number of principal components (PCs) to retain for subsequent dimensionality reduction analyses. The X-axis represents the number of principal components, and the Y-axis represents the standard deviation corresponding to each PC. The scatter points in the figure illustrate the trend of change between PCs and their corresponding standard deviations. (**c**) Unsupervised clustering of 28 cell clusters. (**d**) Expression of marker genes in the 28 clusters. (**e**,**f**) Annotation of nine cell subpopulations (fibroblasts, T cells, macrophages, etc.). (**g**) Proportion of the nine subpopulations in the endometriosis and control samples. (**h**) Key immune cells differ between endometriosis and control samples. Ns not significant, ** *p* < 0.01.

To identify additional cell populations associated with endometriosis, scRNA-seq analysis was performed. After quality control, a total of 151,571 cells and 23,767 genes were retained from the dataset (Figure S2), and the top 2000 highly variable genes were identified (Figure S3). PCA revealed no significant outliers (Figure 5a), and 28 PCs were selected for subsequent analysis based on the elbow plot (Figure 5b). Unsupervised clustering then defined 28 distinct cell clusters (Figure 5c), and the expression profiles of marker genes across these clusters were examined (Figure 5d). Based on these results, nine cell subpopulations, including fibroblasts, T cells, and macrophages, were annotated (Figure 5e,f; Table 5). The proportions of the nine cell subpopulations in endometriosis and control samples were compared (Figure 5g). Macrophages and stromal stem cells showed significant differences between groups (*p* < 0.05) (Figure 5h). Specifically, macrophages were more abundant in endometriosis samples, whereas stromal stem cells were more prevalent in controls. Therefore, macrophages and stromal stem cells were designated as key cells for further analyses.

### 3.7. Functional Analysis of Key Cells

**Figure 6:**
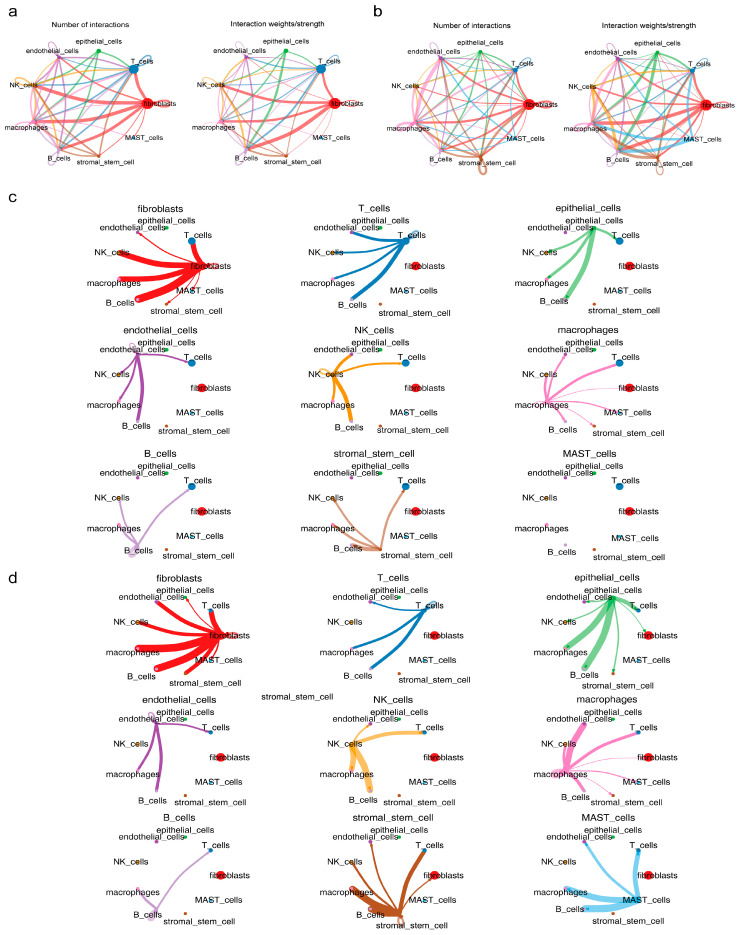
Cellular communication analysis of key cells. (**a**,**b**) Cellular communication analysis of macrophages and stromal stem cells. Lines of different colors represent the interaction pairs between different cells involved in cell–cell communication strength or count; the thicker the line, the stronger the communication strength or the greater the communication count. Points of different colors represent cell types; the larger the circle, the greater the number of cells. (**c**,**d**) Interactions of macrophages with endothelial cells and stromal stem cells with fibroblasts and endothelial cells in endometriosis samples. A line of one color corresponds to the interactions between one cell type and all other cell types—for example, red represents the interactions between fibroblasts and all other cells.

To elucidate the functions of key cells, cellular communication and pseudo-time analyses were conducted. Cellular communication analysis revealed that macrophages exhibited reduced self-communication in endometriosis samples, while stromal stem cells displayed no self-communication compared with control samples (Figure 6a,b). Moreover, macrophage interactions with endothelial cells were weakened in endometriosis samples, and stromal stem cells showed no interactions with fibroblasts or endothelial cells in endometriosis compared with those in controls (Figure 6c,d).

**Figure 7:**
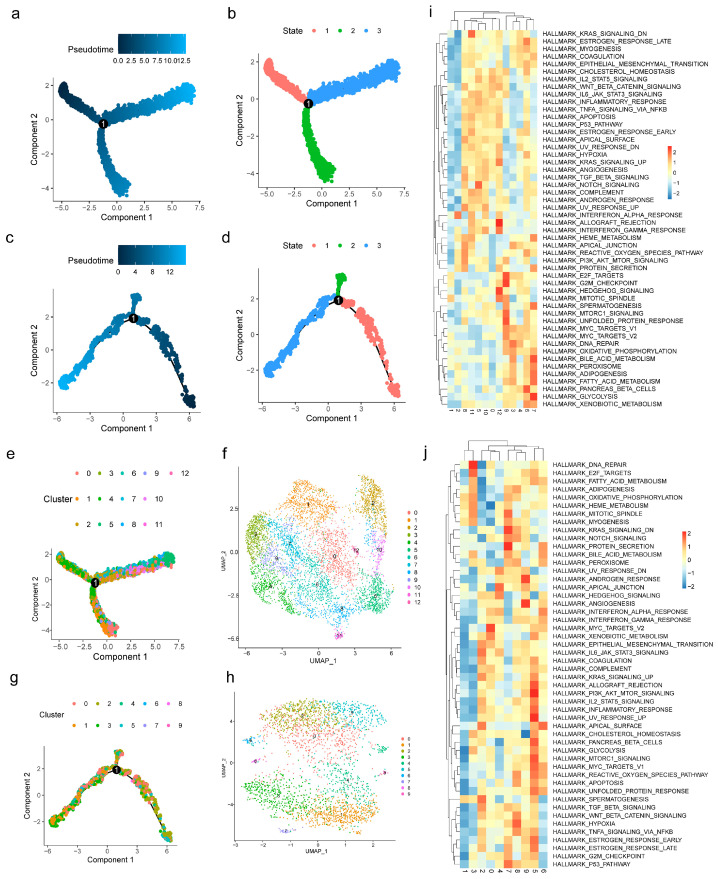
Pseudo-time analysis of key cells. (**a**,**b**) 3 stages of differentiation of macrophages. (**c**,**d**) 3 stages of differentiation of stromal stem cells. (**e**,**f**) 13 subtypes of macrophages. (**g**,**h**) 10 subtypes of stromal stem cells. (**i**) Heat maps of GSVA for pathways of macrophages. (**j**) Heat maps of GSVA for pathways of stromal stem cells.

Pseudo-time analysis demonstrated that both macrophages (Figure 7a,b) and stromal stem cells (Figure 7c,d) progressed through three differentiation stages, with stage 1 representing the initiation stage. Macrophages (Figure 7e,f) and stromal stem cells (Figure 7g,h) were subsequently classified into 13 and 10 cell subtypes, respectively. GSVA showed that macrophage subtypes were enriched in glycolysis (subtype 7), G2M checkpoint (subtype 9), and allograft rejection (subtype 12) (Figure 7i). Subtypes of stromal stem cells were enriched in MYC targets V2 (subtype 0), DNA repair (subtype 3), UV response up (subtype 5), protein secretion (subtype 7), and angiogenesis (subtype 9) (Figure 7j). Several pathways enriched in these subtypes, such as glycolysis and protein secretion, were associated with endometriosis. These findings suggested that key cells may contribute to endometriosis development through the regulation of such pathways.

### 3.8. BMP6 and SLC48A1 Were Verified by RT-qPCR

**Figure 8:**
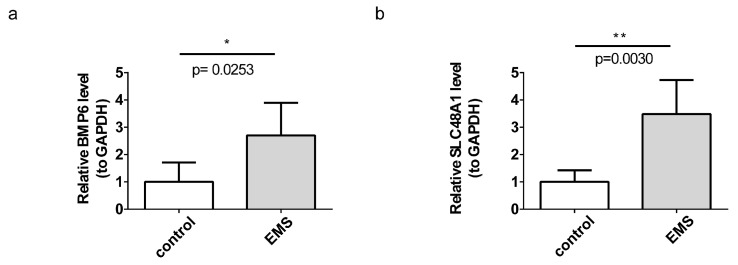
RT-qPCR validation of *BMP6* and *SLC48A1*. * *p* < 0.05. (**a**) Expression of *BMP6* in endometriosis and control samples. (**b**) Expression of *SLC48A1* in endometriosis and control samples. ** *p* < 0.01.

To validate the findings from bioinformatics analyses, RT-qPCR was performed for *BMP6* and *SLC48A1* in all endometriosis and control samples. The RT-qPCR results were consistent with the bioinformatics analyses, confirming that *BMP6* and *SLC48A1* were differentially expressed between the endometriosis and control groups. Both genes exhibited significantly higher expression in endometriosis samples (Figure 8a,b).

## 4. Discussion

The role of ferroptosis in endometriosis affects the survival and clearance of ectopic tissues. However, its underlying mechanisms remain controversial [55]. Iron metabolism, a key metabolic pathway linked to ferroptosis in endometriosis pathogenesis, has been widely recognized [56]. Iron metabolites, particularly iron and ferritin in cyst fluids and endometriotic tissues, act as biomarkers associated with the pathophysiological and pathogenic processes of ovarian endometriosis [57]. Abnormalities in iron metabolism, as revealed in endometriosis peritoneal fluid, are correlated with increased red blood cell counts and elevated hemoglobin levels. Iron homeostasis contributes to endometriosis pathogenesis primarily through hemoglobin by-products and inflammatory or oxidative stress [58]. The investigation of iron metabolism–related biomarkers in endometriosis offers potential for early diagnosis and therapeutic prediction. Through differential expression analysis, MR analysis, receiver operating characteristic curve analysis, and expression level assessment, two IM-RGs were identified as potential biomarkers for endometriosis. Moreover, single-gene GSEA, GeneMANIA, ceRNA network, immune infiltration, and single-cell analyses revealed the biological pathways, molecular mechanisms, regulatory networks, and expression patterns of *BMP6* and *SLC48A1* in macrophages and stromal cells, thereby providing valuable insights into endometriosis diagnosis and treatment strategies.

The biomarkers *BMP6* and *SLC48A1* demonstrated significantly elevated expression in endometriosis samples, a finding consistently validated through both bioinformatics and RT-qPCR analyses. The novelty of this study lies in the integration of single-cell transcriptomics with MR to identify iron metabolism–related genes, an approach not previously reported. Moreover, *BMP6* and *SLC48A1* represent newly identified target genes, as they have not been documented in prior studies.

BMP6, a member of the transforming growth factor-β (TGF-β) superfamily, is a multifunctional cytokine involved in diverse biological processes, including the regulation of cell proliferation, differentiation, apoptosis, immune responses, and iron homeostasis [59,60]. Its potential mechanisms of action in endometriosis are likely multifaceted. Firstly, as a key regulator of iron metabolism, BMP6 may respond to iron overload caused by recurrent bleeding within ectopic lesions, upregulating hepcidin expression via the canonical SMAD signaling pathway and thereby disrupting both systemic and local iron homeostasis [60,61]. This abnormal accumulation of iron can catalyze excessive production of reactive oxygen species (ROS), leading to oxidative stress that promotes inflammation, cell proliferation, and tissue fibrosis, collectively creating a microenvironment favorable for the survival and growth of ectopic endometrial tissue [62,63]. Moreover, as a member of the TGF-β superfamily, BMP6 may directly influence endometrial cells through autocrine or paracrine signaling by engaging BMP receptors on the cell surface, thereby activating downstream pathways that enhance proliferation, invasiveness, and resistance to apoptosis [59]. Previous studies also suggest that BMP6 modulates granulosa cell function, affects steroid hormone synthesis, and participates in neutrophil accumulation and regulation within the ovary, potentially promoting endometriosis progression through crosstalk with estrogen signaling [64,65]. Collectively, these observations suggest that BMP6 contributes to endometriosis development and progression through multiple interconnected mechanisms, including regulation of iron metabolism, direct cellular signaling, and interactions with hormonal pathways, underscoring its potential as a therapeutic target.

In addition, this study found that SLC48A1 is upregulated in endometriosis and may drive disease progression by modulating iron metabolism. SLC48A1 encodes the heme transporter HRG-1, responsible for transferring heme iron from lysosomes to the cytoplasm for reutilization [66]. In ectopic lesions, cyclical bleeding results in massive red blood cell extravasation, releasing heme as a substantial iron source [67]. Upregulation of SLC48A1 likely enhances the capacity of endometrial cells to recycle heme iron, causing intracellular iron overload [68]. Excess free iron catalyzes the generation of ROS, leading to oxidative stress that not only directly promotes cell proliferation, invasion, and resistance to apoptosis but also activates inflammatory signaling and fibrotic processes, thereby establishing a microenvironment favorable for ectopic endometrial survival and growth [69,70,71]. Therefore, SLC48A1 may act as a critical molecular link connecting lesion hemorrhage to disease progression via the heme iron recycling–iron overload–oxidative stress axis, representing a potential therapeutic target in endometriosis.

The impaired immune system, characterized by abnormal activities of neutrophils, macrophages, NK cells, and dendritic cells that secrete cytokines and defensins, has been recognized as a major factor in the initiation of endometrial lesions, primarily through angiogenesis, growth, and invasion of endometriosis cells [72]. In this study, M2 macrophages and monocytes were significantly elevated in endometriosis samples. *BMP6* exhibited the strongest negative correlation with monocytes, whereas *SLC48A1* demonstrated the strongest positive correlation with activated NK cells, suggesting their involvement in endometriosis through immune cell regulation. Peritoneal macrophages represent the predominant immune cell population within peritoneal fluid and play a central role in establishing endometriotic lesions, thereby contributing substantially to disease progression [73]. Macrophages located in close proximity to endometriotic cells regulate homeostasis within the immune microenvironment of endometriosis. Notably, macrophage polarization is essential for controlling the initiation and progression of ectopic endometrial cells, with M2 macrophages being strongly implicated in promoting endometriosis development [74]. Moreover, macrophage-derived IL-33/ST2 has been shown to inhibit ferroptosis in endometriosis through the ATF3/SLC7A11 axis [75], indicating that macrophages may influence endometriosis ferroptosis via SLC regulation.

Monocytes and NK cells influence the progression of endometriosis by participating in inflammatory networks that further drive disease development [76]. NK cells present in peritoneal fluid, characterized by CD16 and CD56 expression together with inhibitory and activating receptors, normally function to eliminate endometrial cells during retrograde menstruation. However, in women with endometriosis, alterations in these receptors and cytokine secretion by NK cells contribute to the initiation and progression of the disease [77]. Therefore, endometrial lesions that persist outside the uterus provoke sustained inflammatory responses by continuously recruiting immune cells to ectopic sites. Therefore, endometriosis may be regarded as an autoimmune disorder.

Genetic and epidemiological evidence indicate that endometriosis affects both physical and mental health, with its associations with depression, anxiety, and eating disorders persisting independently of chronic pain, suggesting that additional biological mechanisms may contribute to these relationships [78]. Clinicians should be aware of the high prevalence of anxiety, depression, and sexual dysfunction in endometriosis patients, as early diagnosis and appropriate management may mitigate related psychological comorbidities [2]. Dysregulated iron metabolism, particularly abnormalities in iron regulatory proteins, has been considered a key pathophysiological mechanism underlying multidimensional psychiatric disorders [79]. For example, astrocytes regulate brain iron homeostasis by modulating ferritin heavy chain (Fth1) and ferritin light chain (Ftl1) mRNA distribution. With aging, the Fth1/Ftl1 ratio increases, accompanied by a shift of Fth1 to fine processes, whereas in Alzheimer’s disease, this ratio decreases, redistributing Fth1 to the soma and Ftl1 to large processes near amyloid beta (Aβ) deposits [80]. This study identified iron metabolism–related genes as potential biomarkers for endometriosis. Given the established association between iron metabolism and psychiatric disorders, further investigation is warranted to determine whether these genes may provide mechanistic insights into the psychiatric comorbidities observed in endometriosis.

Although this study is primarily exploratory at the basic research level, our findings may provide meaningful implications for the clinical management of endometriosis. First, the detection of BMP6 and SLC48A1 expression holds promise as a laboratory-based auxiliary diagnostic tool. Monitoring alterations in these genes could help to assess disease activity or therapeutic responsiveness in affected patients. Second, both genes may serve as potential molecular targets for the development of novel therapeutic strategies. For instance, designing small-molecule inhibitors against SLC48A1 could disrupt the iron recycling pathway within ectopic lesions, thereby restraining their growth. Future investigations should aim to validate the diagnostic relevance of these genes in larger clinical cohorts and to evaluate the feasibility of targeting them for therapeutic intervention.

In summary, this study identified two iron metabolism–related biomarkers in endometriosis through bioinformatics analysis, providing a novel theoretical basis for further mechanistic research. Nevertheless, several limitations should be acknowledged. First, the analyses were primarily dependent on publicly available databases, which may restrict the generalizability of the findings. Second, experimental validation was limited to RT-qPCR, and the reliance on a single technique may compromise accuracy. To address these issues, we plan to collect multicenter, prospective clinical cohorts encompassing a broader range of populations and disease subtypes, in order to validate the robustness of our observations and construct a more reliable diagnostic model. Moreover, to strengthen the rigor of validation, we intend to incorporate multiple technical platforms, such as Western blotting and immunohistochemistry, to confirm biomarker expression at the protein level. In addition, functional gain- and loss-of-function studies in cellular and animal models will be performed to elucidate the underlying molecular mechanisms, thereby enhancing both the reliability and the scientific value of our conclusions.

## 5. Conclusions

This study integrated MR and transcriptome data analyses to investigate ferroptosis-related biomarkers in endometriosis, preliminarily revealing the potential functions of these biomarkers in the disease and their associations with immune cells. These findings provided a foundation for further exploration of the molecular mechanisms of iron metabolism–related genes in endometriosis and also offered new insights for the early diagnosis and development of therapeutic strategies for the disease.

## Figures and Tables

**Table 1:** Causal relationship between exposure factor and outcome.

Outcome	Exposure	Method	nSNP	*p* Value	or	or_lci95	or_uci95
ukb-b-10903	eqtl-a-ENSG00000153162 (*BMP6*)	Inverse variance weighted	10	0.04750698	1.001002221	1.000010995	1.001994429
ukb-b-10903	eqtl-a-ENSG00000211584 (*SLC48A1*)	Inverse variance weighted	5	0.046119158	1.000723176	1.000012434	1.001434424

**Table 2:** Heterogeneity test of MR.

Symbol	Exposure	Method	Q	Q_df	Q_*p* Value
*SLC48A1*	eqtl-a-ENSG00000211584	Inverse variance weighted	1.048	4.000	0.902
*BMP6*	eqtl-a-ENSG00000153162	Inverse variance weighted	10.915	9.000	0.282

**Table 3:** Horizontal pleiotropy test of MR.

Symbol	Exposure	Egger_Intercept	se	*p* Value
*BMP6*	eqtl-a-ENSG00000153162	0.000323385	0.000198411	0.141775357
*SLC48A1*	eqtl-a-ENSG00000211584	4.52 × 10^−5^	0.000131705	0.753939952

**Table 4:** Steiger test analysis of MR.

Exposure	Symbol	snp_r2.exposure	snp_r2.outcome	Correct_Causal_Direction	Steiger_*p* Value
eqtl-a-ENSG00000153162	*BMP6*	0.031483363	3.22 × 10^−5^	TRUE	1.63 × 10^−194^
eqtl-a-ENSG00000211584	*SLC48A1*	0.042311398	1.09 × 10^−5^	TRUE	1.11 × 10^−268^

**Table 5:** Cell subpopulations annotated.

Cell Clusters	Cell Subpopulations
0, 1, 2, 4, 9, 10, 13, 16, 17, 27	fibroblasts
3, 5, 7, 12, 15	T cells
8, 20, 23	endothelial cells
6, 22	epithelial cells
11, 26	NK cells
14, 21, 24	macrophages
18	B cells
19	stromal stem cell
25	MAST cells

## Data Availability

The original contributions presented in this study are included in the article/supplementary material. Further inquiries can be directed to the corresponding author.

## Supplementary Materials

The following supporting information can be downloaded at: https://www.mdpi.com/article/10.3390/cimb47100831/s1.

## Abbreviations

The following abbreviations are used in this manuscript:

ADAMTS13	A Disintegrin and Metalloproteinase with Thrombospondin Motifs 13
AHSG	Alpha-2-HS-Glycoprotein
ATF3/SLC7A11	Activating Transcription Factor 3/Solute Carrier Family 7 Member 11
BP	Biological process
BMP	Bone Morphogenetic Protein
BMP4	Bone Morphogenetic Protein 4
BMP6	Bone Morphogenetic Protein 6
BPs	Biological Processes
CCs	Cellular Components
CD16	Cluster of Differentiation 16
CD56	Cluster of Differentiation 56
cDNA	Complementary DNA
ceRNA	Competing endogenous RNA
CHRDL2	Cysteine-Rich Dipeptidase-Like 2
CI	Confidence Interval
CIBERSORT	Cell-type Identification By Estimating Relative Subsets Of RNA Transcripts
DEGs	Differentially expressed genes
DEIM-RGs	Differentially Expressed IM-RGs
DNA	Deoxyribonucleic Acid
EC	Ectopic Endometrium
eQTL	Expression Quantitative Trait Loci
EU	Eutopic endometrium
FC	Fold change
FDR	False Discovery rate
GAPDH	Glyceraldehyde-3-Phosphate Dehydrogenase
GeneMANIA	Gene Microarray Analysis Network and Interaction Analysis
GEO	Gene expression omnibus
GGI	Gene-gene Interaction
GO	Gene ontology
GPL	Gene Expression Omnibus Platform
GSE	Gene Expression Series
GSEA	Gene set enrichment analysis
GSVA	Gene set variation analysis
GWAS	Genome-wide association study
HRG-1	Heme Responsive Gene 1
IEU	Integrative Epidemiology Unit
IL-33/ST2	Interleukin-33/Suppressor of Tumorigenicity 2
IM-RGs	Iron Metabolism-related Genes
IV	Instrumental Variable
IVs	Instrumental Variables
IVW	Inverse Variance Weighted
KEGG	Kyoto encyclopedia of genes and genomes
LOO	Leave-one-out
LD	Linkage Disequilibrium
MFs	Molecular Functions
MR	Mendelian Randomization
MSigDB	Molecular Signatures Database
MYC	Myelocytomatosis Oncogene
OR	Odd Ratio
NEAT1	Nuclear Enriched Abundant Transcript 1
NK	Natural Killer
PC	Principal Component
PCA	Principal Component Analysis
PINK1	PTEN-Induced Kinase 1
RNA	Ribonucleic Acid
ROC	Receiver operating characteristic
RT-qPCR	Reverse transcription-quantitative polymerase chain reaction
scRNA-seq	Single-cell RNA Sequencing
SLC	Solute Carrier Family
SLC48A1	Solute Carrier Family 48 Member 1
SNPs	Single Nucleotide Polymorphisms
TGFβ	Transforming Growth Factor Beta
UV	Ultraviolet radiation
