# Supplementary material for: Identification and Validation of Iron Metabolism-Related Biomarkers in Endometriosis: A Mendelian Randomization and Single-Cell Transcriptomics Study
Source: Curr Issues Mol Biol. 2025 Oct 9;47(10):831. doi: 10.3390/cimb47100831 (PMC12564322; doi:10.3390/cimb47100831)
Supplement: Supplementary file 1 [file cimb-47-00831-s001.zip › Table S3.pdf]

Table S3 Primer sequences of biomarkers

primers	**sequences**
BMP6 F	CCTTCCCATCCTTTCTGCGA
BMP6 R	ATGCCAGTCTATTCCGCGAG
SLC48A1 F	GGTCAGAGAATGGCCCTAGT
SLC48A1 R	CCTCCCTCTTCGAAAGCCAA
internal reference-GAPDH F	CGAAGGTGGAGTCAACGGATTT
internal reference-GAPDH R	ATGGGTGGAATCATATTGGAAC

Note: F (Forward Primer) is the upstream primer, and R (Reverse Primer) is the downstream primer.
