# Supplementary material for: Identification and Validation of Iron Metabolism-Related Biomarkers in Endometriosis: A Mendelian Randomization and Single-Cell Transcriptomics Study
Source: Curr Issues Mol Biol. 2025 Oct 9;47(10):831. doi: 10.3390/cimb47100831 (PMC12564322; doi:10.3390/cimb47100831)
Supplement: Supplementary file 1 [file cimb-47-00831-s001.zip › Table S2.pdf]

Table S2 Marker genes of different cell types

| cell clusters | marker genes |
|---|---|
| epithelial cells | EPCAM, KRT19, KRT18, KRT5, KRT15 |
| macrophages | CD68, MS4A4A, MS4A7 |
| T cells | CD2, CD3D/E/G |
| MAST cells | CD117, TPSB2, TPSAB1 |
| NK cells | TRDC, KLRC1 |
| neutrophils | CD16, CD66 |
| endothelial cell | AQP1, MYCT1, CDH5, PECAM1 |
| fibroblasts | COL1A1, COL3A1, COL1A2), B cell(IGKC, CD79A, CD19, CD79B |
| stromal stem cell | ITGB1, THY1 |
